# Effect of sorafenib on cisplatin-based chemoradiation in head and neck cancer cells

**DOI:** 10.18632/oncotarget.8275

**Published:** 2016-03-22

**Authors:** Nikolaus Möckelmann, Thorsten Rieckmann, Chia-Jung Busch, Benjamin Becker, Lisa Gleißner, Konstantin Hoffer, Maria Omniczynski, Leonhard Steinmeister, Simon Laban, Reidar Grénman, Cordula Petersen, Kai Rothkamm, Ekkehard Dikomey, Rainald Knecht, Malte Kriegs

**Affiliations:** ^1^ Head and Neck Cancer Center of The University Cancer Center Hamburg (UCCH), Department of Otorhinolaryngology and Head and Neck Surgery, University Medical Center Hamburg-Eppendorf, Hamburg, Germany; ^2^ Head and Neck Cancer Center of The University Cancer Center Hamburg (UCCH), Laboratory of Radiobiology & Experimental Radiooncology, University Medical Center Hamburg-Eppendorf, Hamburg, Germany; ^3^ Department of Otorhinolaryngology – Head and Neck Surgery and Department of Medical Biochemistry and Genetics, Turku University and University Hospital of Turku, Turku, Finland; ^4^ Head and Neck Cancer Center of The University Cancer Center Hamburg (UCCH), Department of Radiotherapy and Radiooncology, University Medical Center Hamburg-Eppendorf, Hamburg, Germany; ^5^ Department of Otorhinolaryngology and Head and Neck Surgery, Ulm University Medical Center, Ulm, Germany

**Keywords:** HNSCC, sorafenib, molecular targeting, radiosensitization, cisplatin

## Abstract

Despite aggressive chemoradiation (CRT) protocols in the treatment of patients with head and neck squamous cell carcinomas (HNSCC), the outcome is still unfavorable. To improve therapy efficacy we had already successfully tested the multikinase inhibitor sorafenib in combination with irradiation (IR) in previous studies on HNSCC cell lines. In this study we investigated its effect on combined CRT treatment using cisplatin.Radio- and chemosensitivity with and without sorafenib was measured in four HNSCC cell lines and normal fibroblasts (NF) by colony formation assay. Apoptosis and cell cycle analysis were performed by flow cytometry.

In HNSCC cells, sorafenib enhanced the antiproliferative effect of cisplatin without affecting apoptosis induction and with only minor effects on cell inactivation. Sorafenib added prior to irradiation enhanced cellular radiosensitivity in three of the tested HNSCC cell lines and caused massive overall cell inactivation when combined with CRT. In contrast, sorafenib did not radiosensitize NF and reduced cisplatin-induced cell inactivation. Cell inactivation by IR and cisplatin is further increased by the addition of sorafenib in HNSCC, but not in NF cells. Therefore, sorafenib is a promising candidate to improve therapy efficacy for HNSCC.

## INTRODUCTION

Most head and neck squamous cell carcinomas (HNSCC) are diagnosed in a locoregionally advanced stage (stage III to IVB), for which a single treatment modality is ineffective. Therefore, these patients are treated with combined therapeutic regimens including surgery, radiation, and chemotherapy. The standard therapy is a platinum-based chemotherapy either given sequentially or concomitant to radiotherapy (RT). Despite this aggressive treatment, tumour recurrence rates are high and the five-year survival rate for these patients is still limited to 30-40%, but adverse side effects lead to reduced compliance rates of CRT treated patients and prohibit further dose escalation [1-6]. In order to improve the efficacy of chemoradiation (CRT) for HNSCC patients, without increasing the side effects on normal tissue, targeted therapeutics have been added to the standard regimens using either monoclonal antibodies (mAb) or small molecule inhibitors in clinical trials.

Besides some alternative strategies, so far the focus of molecular targeting for HNSCC patients has been on the inhibition of the epidermal growth factor receptor (EGFR). However, recent pre-clinical and clinical data suggest that EGFR inhibition might not improve tumor control and response especially to CRT [7, 8]. Therefore new effective strategies have to be evaluated in order to improve the efficacy of CRT treatment without increasing normal tissue damage. Although, the majority of the tested targeted therapeutics could not show an improvement in antitumor activity of the combined treatment with CRT until now [7], some agents are still under clinical investigation as part of trials (NCT01737008, NCT01824823, NCT02131155, NCT00442455, NCT00629226).

In this context the multi-target kinase inhibitor sorafenib seems to be promising. Sorafenib is already FDA approved for the treatment of different tumor entities [9-11]. In HNSCC, sorafenib has only been investigated clinically in the palliative setting without radiotherapy [12, 13]. However, we have already demonstrated, that sorafenib inhibits proliferation, causes approximately 50% of cell inactivation and is able to radiosensitize human papilloma virus (HPV)-negative HNSCC cells effectively [14], which is in line with data from other groups and for other tumor entities [15-18]. Furthermore, we were able to show that radiosensitization is likely mediated by inhibition of DNA double strand break repair [14]. However, the effect of sorafenib on irradiation in combination with cisplatin chemotherapy has not been evaluated so far, either for HNSCC or for normal cells with the latter potentially indicating normal tissue damage.

Since cisplatin-based CRT is the standard treatment for advanced HNSCC, the aim of this study was to investigate the effects of sorafenib on HNSCC and normal cells treated with both irradiation and cisplatin in order to evaluate this combination for further pre-clinical investigations using xenograft tumors.

## RESULTS

### Effect of sorafenib on radiosensitivity

**Figure 1:**
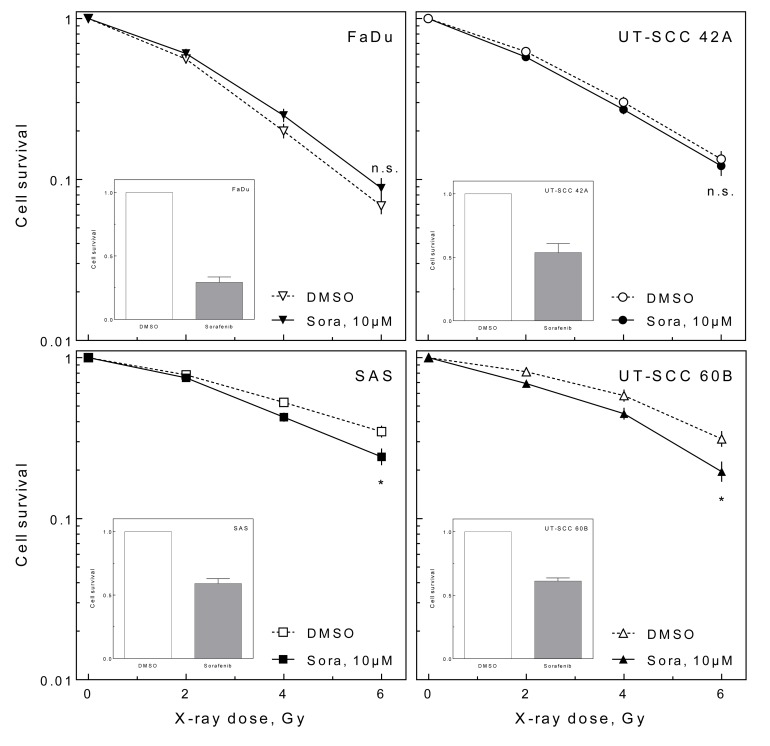
Effect of sorafenib on cellular radiosensitivity Exponentially growing HNSCC cells were irradiated with doses up to 6 Gy with or without 10 μM sorafenib pre-treatment for 2 h. Twenty-four hours later cells were re-plated (delayed plating). The relative radiosensitivity (large graph) and the relative cytotoxic effect of sorafenib alone (small inlayed graphs) were determined using the colony forming assay.

In a previous study we reported a radiosensitizing effect of sorafenib on HNSCC cell lines [14]. We verified this finding by screening a panel of 20 different HNSCC cell lines by colony formation assay under delayed plating conditions resulting in five cell lines which could be sensitized to ionizing radiation by sorafenib (data not shown). For further studies, we chose four cell lines, one which does not become sensitized (FaDu) and three which become sensitized, either to a small (UT-SCC 42A), a moderate (SAS) or a large (UT-SCC 60B) extent (Figure 1). In all cell lines tested, sorafenib additionally caused a cytotoxic effect of approximately 50% (Figure 1 inlays). For FaDu and UT-SCC 42A these responses had been reported previously [14].

### Effect of sorafenib on cisplatin treatment

**Figure 2:**
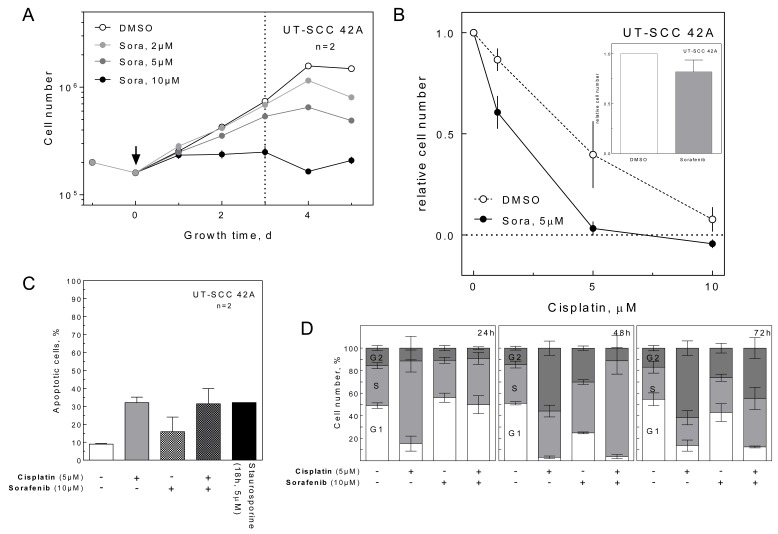
Effect of sorafenib on proliferation, apoptosis and cell cycle **A.** Inhibition of cell proliferation. UT-SCC 42A cells were treated with sorafenib as indicated and the cells were counted daily up to 5 d after sorafenib addition. The absolute cell numbers are depicted. **B.** Enhancement of the anti-proliferative effect of cisplatin. Cells were treated with increasing concentrations of cisplatin with and without 5 μM sorafenib. Three days later, the cell number was quantified, the number of plated cells was subtracted and the numbers were normalized to the untreated control. The fraction of treated cells compared to control (DMSO-treated) cells is depicted. The relative effect of sorafenib alone is given in the inlay. **C.** Induction of apoptosis. Cells were treated with 10 μM sorafenib and 5 μM cisplatin. Shown is the percentage of apoptotic cells after 72 h treatment as measured by flow cytometry. Cells were incubated with 5 μM staurosporine for 18 h as a positive control. **D.** Effect on cell cycle distribution. Cells were treated with 10 μM sorafenib and/or 5 μM cisplatin. Cell cycle distributions were determined by flow cytometry for up to 72 h post treatment.

To test the effect of sorafenib on cisplatin, we analyzed UT-SCC 42A cells in terms of proliferation, apoptosis and cell cycle. Because sorafenib had a dose-dependent antiproliferative effect with maximal growth inhibition at 10 μM (Figure 2A), we used 5 μM sorafenib to analyze cell proliferation in combination with cisplatin. As shown in Figure 2B, cisplatin alone induced a dose-dependent growth inhibition, which was further enhanced by sorafenib. This effect could not be attributed to an increase in cisplatin-induced apoptosis (Figure 2C) or a pronounced cell cycle arrest (Figure 2D), since even 10 μM sorafenib caused only a small additional S-phase delay and G2-phase arrest compared to the samples treated with cisplatin only.

**Figure 3:**
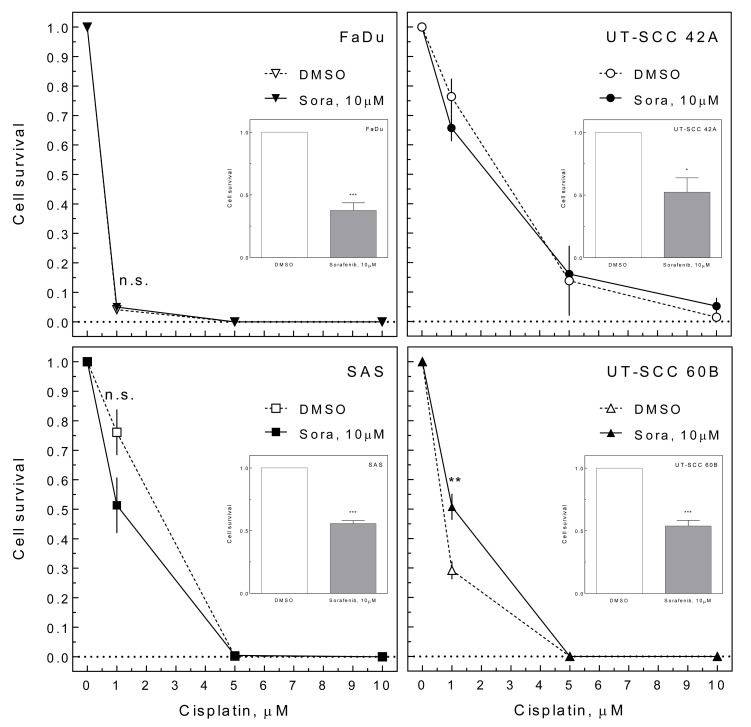
Effect of sorafenib on cisplatin-induced cell inactivation HNSCC cells were treated with 10 μM sorafenib and increasing concentrations of cisplatin for 24 h. The medium was changed and cell inactivation was measured by colony forming assay (pre-plating). The relative cell survival is depicted including the effect of sorafenib treatment alone (inlays).

To test whether enhanced inhibition of proliferation by sorafenib also causes improved cell inactivation in combination with cisplatin, we performed colony formation assays. Therefore cells were treated for 24 h with 10 μM sorafenib and different concentrations of cisplatin (1-10 μM; pre-plating). The effect of cisplatin alone varied, with FaDu cells being very sensitive towards cisplatin while UT-SCC 42A cells were quite resistant (Figure 3). Adding sorafenib caused heterogeneous effects, with UT-SCC 42A and SAS cells becoming slightly more sensitive while UT-SCC 60B cells became significantly more resistant. Like under delayed plating conditions sorafenib caused an additional cytotoxic effect (Figure 3 inlays).

### Effect of sorafenib on combined treatment

To test whether sorafenib influences the interaction of irradiation and cisplatin, we analyzed the cellular radiosensitivity in the presence of either cisplatin or cisplatin and sorafenib (10 μM) under delayed plating conditions. Because of the strong cytotoxic effect of cisplatin we used only 1 μM cisplatin for these experiments.

**Figure 4:**
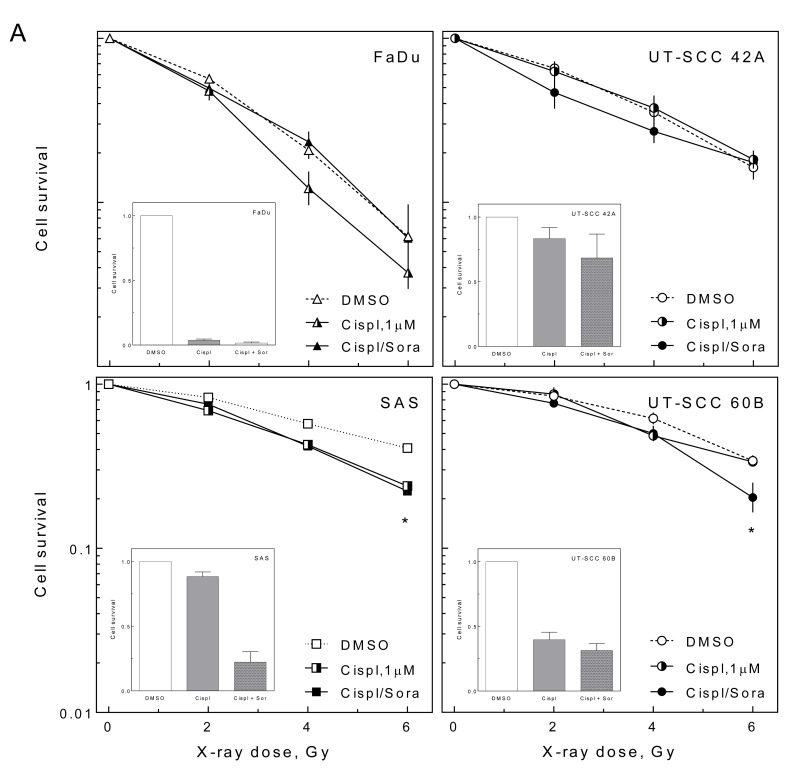

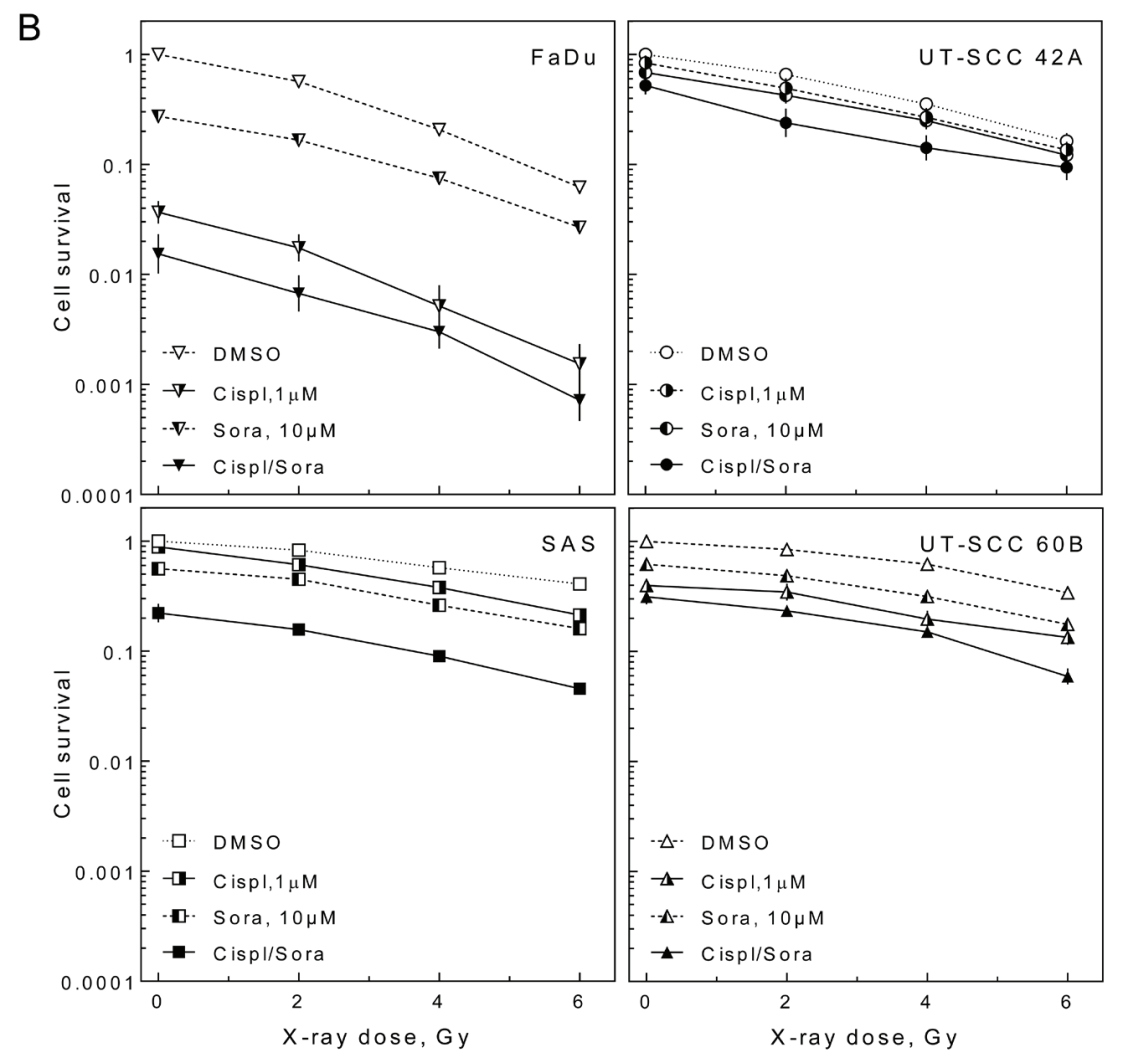
Effect of sorafenib on cell inactivation after combined treatment HNSCC cells were treated with 10 μM sorafenib and 1 μM cisplatin for 2 h before cells were irradiated using different doses. Twenty-four hours later cells were re-plated and cell inactivation was measured by colony forming assay (delayed plating). **A.** Relative cell survival (transformed values) including the effect of cisplatin and cisplatin + sorafenib treatment alone (inlays). **B.** Non transformed values.

Cisplatin caused a radiosensitization in three of the cells lines (FaDu, SAS, UT-SCC 60B) (Figure 4A). This sensitization was abrogated by sorafenib in FaDu cells but was not affected in SAS and UT-SCC 60B cells. Furthermore, sorafenib caused a slight but significant sensitization in cisplatin-treated UT-SCC 42A cells at low doses. The effect of cisplatin and sorafenib without IR is depicted in the inlays.

To summarize, sorafenib caused heterogeneous effects when combined with IR and cisplatin. However, when the effects of cisplatin and sorafenib were not normalized to the intrinsic cytotoxicity (non transformed values) it becomes obvious that the triple combination of IR, cisplatin and sorafenib causes a massive cell inactivation. This cell inactivation was always stronger compared to the treatment of HNSCC cells with IR, IR + cisplatin or IR + sorafenib, alone (Figure 4B).

### Effect of sorafenib on normal fibroblasts

**Figure 5:**
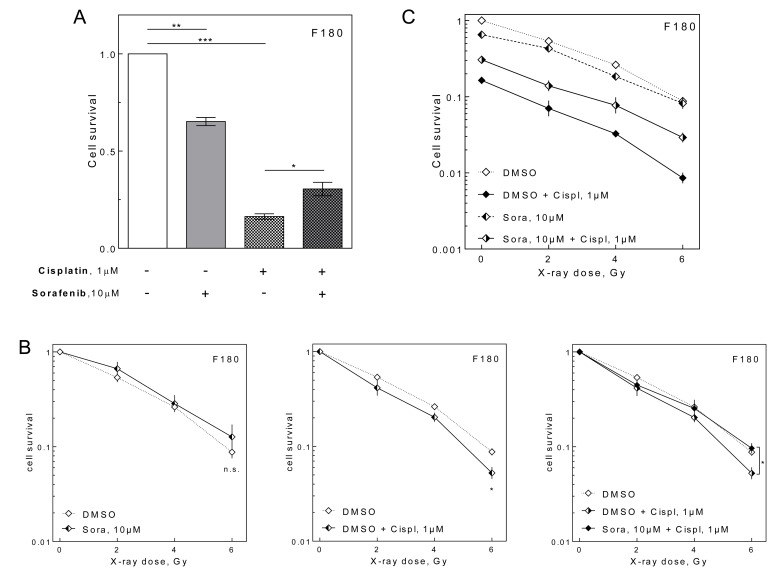
Effect of sorafenib on NF Relative cell survival as measured by colony forming assay using F180 NF treated with 10 μM sorafenib and 1 μM cisplatin as indicated. **A.** Unirradiated cells. **B.** Relative effect of sorafenib (left), cisplatin (center) and combined (right) treatment on radiosensitivity (transformed values). **C.** Non-transformed values.

One limiting parameter for combining tyrosine kinase inhibitors like sorafenib with CRT could be the side effects induced by massive inactivation of normal cells. Therefore, we asked, if addition of sorafenib to cisplatin and irradiation enhances cell inactivation of normal cells in the colony formation assay by treating NF under delayed plating conditions. Although sorafenib alone caused cell inactivation in NF, the cell inactivation induced by cisplatin was significantly reduced when sorafenib was added (Figure 5A). Furthermore, no radiosensitization was observed by sorafenib in NF contrary to HNSCC cells (Figure 5B, left panel). In contrast, cisplatin induced a modest, yet significant radiosensitization (Figure 5B, central panel). Strikingly this was reverted by the addition of sorafenib (Figure 5B, right panel). All together the sorafenib treatment resulted in an improved cell survival of irradiated and cisplatin-treated NF as shown by Figure 5C, displaying non transformed values.

## DISCUSSION

In this study we investigated the effect of sorafenib on HNSCC and normal cells treated with both irradiation and cisplatin in order to evaluate this combination for further pre-clinical and potential clinical investigations.

We could demonstrate that sorafenib enhances radiosensitivity, which is in line with our previous data [14]. This radiosensitization can be observed in approximately a quarter of the HPV-negative HNSCC cell lines tested (data not shown), indicating that this is a frequent phenomenon. Furthermore, this sensitization was observed under experimental conditions (delayed plating) in which, for example, EGFR inhibitors failed to sensitize various tumor cell lines [8, 19]. As delayed plating results are thought to better reflect tumor cell killing *in vivo*, the observed radiosensitization is expected to translate into improved tumor control [8].

In combination with cisplatin we observed heterogeneous responses ranging from sensitization (SAS) to resistance (UT-SCC 60B). Nevertheless, these effects were quite small and when sorafenib was added to chemoradiation treatment, all cell lines displayed improved overall cell inactivation, regardless of any radio- or chemosensitization (Figure 4B). Therefore, sorafenib is a promising candidate for combined targeted treatment of HNSCC which warrants further investigation.

In clinical practice, cisplatin is added to ionizing radiation because of its clinically observed radiosensitizing effect, reflected by higher survival and response rates of HNSCC patients treated with CRT *versus* RT alone [2, 3]. In this study we observed cellular radiosensitization by cisplatin in three out of four HNSCC cell lines. This sensitization was diminished by sorafenib in FaDu cells. However, as mentioned above, reduced overall cell survival could be observed in all triple-treated samples compared to the cisplatin and IR-treated samples. This argues for an addition of sorafenib to CRT even for cells / tumors which are not chemo- or radiosensitized by sorafenib. This is of relevance since some clinical trials combining targeted therapeutics and CRT could show even a lower antitumor activity, albeit not statistically significant, for patients receiving the targeted agent in addition to CRT [5, 20, 21].

To improve treatment outcome for HNSCC patients distinct tyrosine kinase inhibitors (TKI) in combination with cisplatin-based CRT are under extensive clinical investigation (NCT01737008, NCT01427478, NCT01824823, NCT02131155, NCT00442455, NCT00629226). However, different TKI tested in combination with cisplatin and RT, namely erlotinib, gefitinib and lapatinib, failed to show an improvement in survival but caused increased toxicity in most trials [22-27]. Our data now suggest that sorafenib might improve tumor control by improving tumor cell inactivation. However, until now, no clinical data exist on the effects of sorafenib in the combined treatment with cisplatin-based CRT in HNSCC. One planned clinical trial investigating the triple combination has been withdrawn before enrollment (NCT00627835). There is data from single arm trials on the efficacy of sorafenib monotherapy and the combined treatment with alkylating agents, such as carboplatin and cisplatin, in recurrent/metastatic HNSCC (RM-HNSCC) [12, 13, 28-30]. In summary, sorafenib combined with cisplatin seems to have an encouraging efficacy profile with tolerable toxicity in most studies. However, a severe risk for side effects such as hand-foot syndrome (HFS) and myelosuppression was observed in the above trials. Therefore, these adverse effects must be taken into consideration in the application of sorafenib. On a cellular level we could show here that sorafenib protected normal cells from inactivation by cisplatin. Whether this translates to normal tissue protection in an *in vivo* setting remains to be seen.

All together these data provide evidence that sorafenib is a promising targeted agent that could potentially be added to cisplatin-based CRT, as it may make treatment both more effective and less toxic.

## MATERIALS AND METHODS

### Substances

Sorafenib (tyrosine-kinase inhibitor, Nexavar^®^, Bayer HealthCare, Leverkusen, Germany), cisplatin (alkylating agent, Medac, Wedel, Germany), staurosporine (Calbiochem/Merck, Darmstadt, Germany), colcemid (Merck, Darmstadt, Germany), DMSO (vehicle; Roche), propidium iodide (Merck, Darmstadt, Germany), RNase A (Serva, Heidelberg, Germany).

### Cell culture

HPV-negative HNSCC cell lines UT-SCC 42A and UT-SCC 60B were obtained from Reidar Grénman (University of Turku, Finland). HNSCC cells and normal human fibroblasts (NF) F180 were grown in Dulbecco's Modified Eagle's Medium (Life technologies, Carlsbad, CA, USA) containing 10% fetal bovine serum (Biochrome AG, Berlin, Germany) and 4 mM glutamine (Life technologies, Carlsbad, CA, USA) at 37°C, 10% CO_2_ and 100% humidification. HNSCC cell lines were authenticated by short tandem repeat analysis (Department of Human Genetics, UKE, Hamburg, Germany).

### Irradiation (IR)

Cells were irradiated at room temperature with a single dose with 200 kV X-rays (Gulmay RS225, Gulmay Medical Ltd., Byfleet, UK; 15 mA, 0.8 mm Be + 0.5 mm Cu filtering; dose rate of 1.2 Gy/min).

### Proliferation

For proliferation assays, 1×10^5^ cells were seeded into T24 culture flasks. Twenty-four hours later they were treated with different concentrations of sorafenib, dissolved in DMSO or a combined treatment of sorafenib (5μM) and different concentrations of cisplatin. Cell numbers were determined at the indicated time points.

### Cell survival

Cell survival was measured by colony formation. To analyze the cisplatin-sensitivity 500-1000 cells were seeded into T25 culture flasks and were treated with cisplatin, sorafenib or a combination of both 24 h later and the medium was exchanged 24 h later (pre-plating). To analyze radiosensitivity, exponentially growing cells were treated with cisplatin, sorafenib or a combination two hours before irradiation. Cells were harvested and re-plated (500-1000 cells) 24 h after irradiation (delayed plating). NF were re-plated using AmnioMax C-100 Basal Medium (Life technologies, Carlsbad, CA, USA) containing 10% FCS and C-100 supplement (Life technologies, Carlsbad, CA, USA) to optimize colony formation. Cells were allowed to grow until colonies reached equal size, fixed with 70% ethanol and stained with crystal violet. Colonies of more than 50 cells were counted by an observer who was blinded to treatment. Unless indicated otherwise, absolute numbers were normalized to the unirradiated controls (transformed values).

### Apoptosis

For the detection of apoptosis, caspase activity (caspase-1, -3, -4, -5, -6, -7, -8 and -9), was analyzed by flow cytometry 72 h after cisplatin and sorafenib treatment using the Carboxyfluorescin FLICA Apoptosis Detection Kit Caspase Assay (Immunochemistry Technologies, LLC, Bloomington, MN, USA), according to the manufacturer's protocol. Staurosporin served as a positive control. Sorafenib and cisplatin were removed 24 h after treatment by medium exchange.

### Cell cycle

Exponentially growing cells were treated with cisplatin, sorafenib or a combination of both. Sorafenib and cisplatin were removed 24 h after treatment by medium exchange and cells were harvested 0 h, 24 h and 48 h thereafter. Cells were fixed by 70% ethanol, washed with PBS (0.1% Tween) and the DNA was stained with propidium iodide (PI, 10 μg/ml) containing RNase A (RNase A 0.1 μg/ml) for 30 min at room temperature. DNA histograms were constructed using flow cytometry (FACS Scan Canto and FACSDiva software, BD Biosciences, Franklin Lakes, NJ, USA) and the fraction of G1, S and G2 phase cells was calculated using ModFit LT™ software (Verity Software House, Inc., Topsham, ME, USA).

### Data evaluation

Unless indicated otherwise, experiments were repeated at least three times (*n* = 3). The data are presented as mean values (±SEM). GraphPad Prism 6 (GraphPad Software, Inc., La Jolla, CA, USA) was used for analyzing and graphing the data. Student's *t*-test was performed for the statistical analysis. *p*-values were calculated using unpaired two sided tests (* *p* < 0.05; ** *p* < 0.01; *** *p* < 0.001).
